# Correlation Between Balance, Agility, and Speed Among Children Participating in Roller Skating

**DOI:** 10.7759/cureus.116622

**Published:** 2026-09-21

**Authors:** Shivanjali Jadhav, Smita C Patil, Neha Bhosale

**Affiliations:** 1 Department of Sports Physiotherapy, Krishna College of Physiotherapy, Krishna Vishwa Vidyapeeth (Deemed to be University), Karad, IND

**Keywords:** agility t test, arrowhead change of direction speed test, corelation, speed, y balance test

## Abstract

Introduction: Balance, agility, and speed are fundamental motor skills essential for optimal performance in roller skating; understanding their relationship helps identify key performance factors. Early identification of deficits in any of these areas can aid in injury prevention, especially in developing children. This study addresses a gap in the existing literature by examining the interrelationship between these skills in child roller skaters.

Methods: A total of 63 subjects who fulfilled the inclusion and exclusion criteria were selected using convenience sampling. Demographic data were obtained. Balance, speed, and agility were assessed using the Y-Balance Test, Arrowhead Change of Direction Speed Test, and T-test, respectively.

Results: A significant moderate positive correlation was found between agility and speed (r = 0.5011, p < 0.0001). Significant negative correlations were observed between balance and agility for the right lower limb (r = -0.3225, p = 0.0099) and between balance and speed for both the right lower limb (r = -0.3887, p = 0.0016) and left lower limb (r = -0.3091, p = 0.0137). No significant correlation was found between left lower limb balance and agility (r = -0.2139, p = 0.0923).

Conclusion: Dynamic balance is significantly associated with agility and speed in children participating in roller skating. Better balance was related to superior agility and speed performance, with right-limb balance showing stronger associations than left-limb balance. These findings highlight the importance of balance in enhancing skating-related performance.

## Introduction

Inline roller skating has experienced a significant resurgence in popularity [1]. It has become an increasingly popular physical activity among young individuals in recent years. Previous research suggests that regular participation in roller skating facilitates overall physical development by improving muscular fitness and joint function throughout the body [2-4]. Due to the dynamic nature of the sport, skaters require well-developed strength and endurance in the upper and lower limbs, strong trunk musculature, and sufficient hip mobility to execute movements effectively and sustain optimal performance [5].

Balance is defined as the ability to maintain the body's center of gravity within its base of support while preserving postural stability. It plays a vital role in athletic performance, particularly during high-speed movements and dynamic activities that require precise control and coordination. Adequate balance enables athletes to perform complex maneuvers efficiently, whereas deficits in balance may negatively affect performance and increase the likelihood of injury. This is especially important in adolescents, as ongoing neuromuscular development may make them more susceptible to balance-related impairments and injury risks [6].

Agility refers to the ability to perform rapid and controlled changes in direction while maintaining balance and coordination. Current perspectives describe agility as a multifaceted motor skill involving rapid body movement, directional changes, and coordinated limb actions in response to movement demands [7]. It is recognized as a key physical quality that should be developed during childhood and adolescence. It is closely associated with visual perception, spatial awareness, dynamic balance, and coordinated movement. The development of agility is a long-term process that progresses gradually as children grow and refine their motor skills [8].

Speed refers to the ability to move the body or an object over a specified distance in the shortest possible time. It represents the rate of movement without considering the direction of travel and is an important component of athletic performance [9,10]. Agility-based activities commonly involve repeated acceleration, deceleration, and rapid changes in direction, requiring athletes to maintain effective postural control throughout movement. During these actions, the body's momentum tends to continue in the original direction due to inertia, making balance an important factor in achieving stability and executing efficient directional changes. Adequate balance enables athletes to control body positioning and transition smoothly between movements. Previous studies conducted among team handball and male basketball players have reported that better dynamic balance is associated with enhanced agility performance [11,12]. Athletes performing multidirectional sprint tasks must maintain balance and stability while decelerating, changing direction, and reaccelerating. Dynamic stability assists in controlling the center of gravity during these movements, contributing to effective and coordinated performance [13].

Agility encompasses a wide range of movements, from quick footwork actions to rapid whole-body directional changes performed at high speeds. Although speed contributes to agility performance, agility is a multifaceted ability that also depends on factors such as coordination, balance, and neuromuscular control. Due to the shared morphological and physiological characteristics underlying speed, acceleration, and agility, researchers have suggested that these physical attributes are closely related and may influence one another [14].

Despite the recognized importance of balance, agility, and speed in sports performance, the available literature regarding their interrelationship remains limited and inconclusive [15]. Moreover, few studies have specifically explored these variables in child roller skaters. This gap in the literature highlights the need for further investigation. Therefore, the present study was conducted to determine the correlation between balance, agility, and speed in inline roller skating among children.

## Materials and methods

A cross-sectional observational correlational study was conducted to determine the relationship between balance, agility, and speed performance among children participating in roller skating. The study was conducted in Karad, Maharashtra, India, over a period of approximately two months. Participants were recruited using a convenience sampling technique.

The study included 63 children aged 6-13 years who participated in roller skating. Participants were recruited according to predefined inclusion and exclusion criteria.

Male and female children aged 6-13 years who participated in roller skating were eligible for inclusion. The exclusion criteria included a history of recent lower-limb injury or surgery, any musculoskeletal deformity, visual or vestibular system problems, and active lower-limb pathology within the preceding six months.

Sample size

The sample size was determined for a correlation-based analysis. Assuming a moderate expected correlation coefficient of 0.35, with a two-tailed significance level of 0.05 and statistical power of 80%, the minimum required sample size was approximately 62 participants. To ensure adequate data for analysis, a total of 63 participants meeting the eligibility criteria were included in the study.

Ethical considerations

Ethical approval was obtained from the Institutional Ethics Committee, Krishna Vishwa Vidyapeeth (Deemed to be University), Karad, prior to commencement of the study (reference number: KVV/IEC/10/2025). The purpose, procedures, potential risks, and benefits of the study were explained to the participants and their parents/guardians. Written informed consent was obtained from the parents/guardians before participation, and the participants were informed about the study procedures.

Data collection procedure

After obtaining informed consent, demographic information, including age and sex, was recorded for each participant. Participants were then assessed for balance, agility, and speed using standardized field-based outcome measures.

Dynamic balance was assessed using the Y-Balance Test (YBT). The test was performed according to the standardized protocol, with participants maintaining a single-leg stance while reaching with the contralateral limb in the anterior, posteromedial, and posterolateral directions. The reach distances were recorded for each direction and used to determine the participant's balance performance. The YBT has demonstrated good reliability in younger populations and has been reported to provide reliable measures of dynamic balance [16].

Agility was assessed using the T-test. The test required participants to move rapidly through a predefined T-shaped course involving forward running, lateral movement, and backward running. The time taken to complete the course was recorded using a stopwatch, with a lower completion time indicating better agility [17].

Speed was assessed using the Arrowhead Change-of-Direction Speed Test. The test involved sprinting through a predefined course with multiple changes in direction around designated markers. Participants were instructed to complete the course as quickly as possible, and the completion time was recorded. The Arrowhead test has demonstrated acceptable reliability for assessing change-of-direction performance [18].

Prior to testing, participants were familiarized with the testing procedures and were provided with standardized instructions. Adequate rest was provided between trials to minimize the effects of fatigue on performance. All assessments were performed under similar testing conditions, and the best of three trials was considered. Speed and agility were assessed while participants were wearing roller skates.

Confounding factors

Factors that could influence balance, agility, and speed, including age, sex, skating experience, training frequency, and previous lower-limb injury, were considered during participant selection and data collection. Participants with recent lower-limb injury, surgery, deformity, visual or vestibular problems, or active lower-limb pathology were excluded to minimize their potential influence on test performance.

Statistical analysis

The collected data were systematically recorded and analyzed using appropriate statistical methods. Descriptive statistics were used to summarize the demographic and outcome-measure data. The distribution of the data was assessed before performing correlation analysis. Pearson's correlation coefficient was used to determine the relationship between balance, agility, and speed when the assumptions for parametric correlation were satisfied. Statistical significance was set at p < 0.05. The magnitude and direction of the correlation coefficients were interpreted to determine the relationships between the variables.

## Results

Pearson's correlation analysis was performed to determine the relationships between balance, agility, and speed among 63 children participating in roller skating. A statistically significant moderate positive correlation was observed between agility and speed (r = 0.5011, p < 0.0001), indicating that participants with lower agility times also tended to demonstrate higher speed times. The coefficient of determination (r² = 0.2511) indicated that approximately 25.11% of the variance was shared between agility and speed. A significant weak negative correlation was found between agility and YBT Right scores (r = -0.3225, p = 0.0099), suggesting that better dynamic balance on the right limb was associated with improved agility performance. However, the correlation between agility and YBT Left scores was weak and not statistically significant (r = -0.2139, p = 0.0923). Similarly, speed demonstrated a significant negative correlation with both YBT Right (r = -0.3887, p = 0.0016) and YBT Left scores (r = -0.3091, p = 0.0137). These findings indicate that participants with better dynamic balance tended to complete the speed test in less time, reflecting superior performance. Overall, the study findings suggest that dynamic balance is significantly associated with speed and, to a lesser extent, agility among children participating in roller skating. The strongest relationship was observed between agility and speed, while right-limb balance showed stronger associations with performance measures than left-limb balance, as shown in Table 1. 

**Table 1:** Correlation between YBT (right and left), agility and speed YBT: Y-Balance Test.

Variables	Pearson's r	p-value	Strength of correlation	Statistical significance
Agility vs. Speed	0.5011	<0.0001	Moderate positive	Significant
Agility vs. YBT Right	-0.3225	0.0099	Weak negative	Significant
Agility vs. YBT Left	-0.2139	0.0923	Weak negative	Not significant
Speed vs. YBT Right	-0.3887	0.0016	Weak negative	Significant
Speed vs. YBT Left	-0.3091	0.0137	Weak negative	Significant

## Discussion

The present study was conducted to investigate the relationship between balance, agility, and speed among children aged 6-13 years participating in roller skating. The findings revealed a significant moderate positive correlation between agility and speed, along with significant negative correlations between dynamic balance and both agility and speed. These results indicate that children demonstrating better dynamic balance tend to exhibit superior agility and speed performance during roller skating activities. A significant moderate positive correlation was observed between agility and speed (r = 0.5011, p < 0.0001). This finding suggests that children who performed better in agility tasks also demonstrated superior speed performance.

Agility and speed share common neuromuscular and biomechanical components, including rapid force production, lower-limb power, coordination, and efficient movement execution. Little and Williams [15] reported significant relationships between sprint performance and agility measures in young athletes, indicating that speed contributes substantially to change-of-direction ability. The present study demonstrated a significant negative correlation between agility and right-limb dynamic balance (r = -0.3225, p = 0.0099). The negative relationship indicates that better balance performance was associated with lower agility test completion times, reflecting enhanced agility.

Dynamic balance plays a crucial role during rapid directional changes, allowing athletes to control their center of mass while maintaining postural stability. These findings are in agreement with Rokaya et al. [11], who reported that athletes with superior dynamic balance exhibited better agility performance. Similarly, Sterling et al. [12] observed significant associations between balance and agility among basketball players, suggesting that balance contributes to effective movement control during multidirectional tasks. However, no significant correlation was found between agility and left-limb dynamic balance (r = -0.2139, p = 0.0923). The absence of significance may be attributed to limb dominance, individual movement strategies, or asymmetries commonly observed during sports performance.

Previous studies have reported that dominant limb function often demonstrates stronger relationships with athletic performance measures than non-dominant limb function [19]. The findings further revealed significant negative correlations between speed and dynamic balance for both the right limb (r = -0.3887, p = 0.0016) and left limb (r = -0.3091, p = 0.0137). These results suggest that children with better dynamic balance were able to complete speed tasks in shorter durations. Speed performance requires efficient force generation and transmission while maintaining postural control. Dynamic balance contributes to stabilization of the center of gravity during acceleration, deceleration, and directional transitions. These findings support those of Hrysomallis [20], who emphasized the importance of balance in sports performance and reported that athletes with superior balance generally demonstrate enhanced movement efficiency. Similar observations were reported by Pau et al. [21], who highlighted the contribution of balance control to performance.

The stronger correlations observed for right-limb balance compared with left-limb balance may indicate the influence of limb dominance in roller-skating performance. Dominant limbs are frequently utilized for propulsion, stabilization, and skill execution, potentially resulting in greater neuromuscular adaptations [22]. This may explain the stronger association between right-limb balance and both agility and speed outcomes observed in the present study. The findings of this study support the concept that balance serves as a fundamental component underlying athletic performance. Balance facilitates effective force transfer, postural control, coordination, and movement efficiency, all of which contribute to successful execution of agility and speed tasks [6,13,20].

Since roller skating requires continuous maintenance of dynamic stability while performing rapid movements, improvements in balance may positively influence overall skating performance. Overall, the findings indicate that dynamic balance is significantly associated with agility and speed performance among children participating in roller skating. The strongest relationship was observed between agility and speed, while right-limb balance demonstrated stronger associations with performance measures than left-limb balance.

Limitations

The study was limited by its relatively small sample size, wide age range, and recruitment from a restricted geographical area, which may limit the generalizability of the findings. The cross-sectional design also prevents establishing causal relationships between balance, agility, and speed. Variations in participants’ leg dominance, skating experience, training exposure, and physical fitness may have influenced performance and could not be completely controlled.

## Conclusions

Dynamic balance is significantly associated with agility and speed in children participating in roller skating. Better balance performance was associated with improved agility and speed outcomes. A moderate positive correlation was observed between agility and speed, while significant negative correlations were found between balance and both agility and speed. Right-limb balance demonstrated stronger associations with agility and speed than left-limb balance. These findings suggest that dynamic balance is an important contributor to agility and speed performance in young roller skaters.
